# Multistage treatment of industrial ethylene glycol (EG) effluent: integrating chemical extraction, coagulation/precipitation, and decolouration for enhanced wastewater remediation

**DOI:** 10.1038/s41598-026-35153-w

**Published:** 2026-01-28

**Authors:** Ahmed S. Mahmoud, E. Khamis, M. S. Mahmoud, Nouran Y. Mohamed

**Affiliations:** 1 Institute of Environmental studies - Arish University, North Sinai, Egypt; 2 Egyptian Russian University, Badr, Egypt; 3 Chemistry Department, Faculty of Science, Alexandria University, Alexandria, Egypt; 4 Sanitary and Environmental Institute (SEI), Housing and Building National Research Center (HBRC), Cairo, Egypt

**Keywords:** Industrial effluent, Circular economy, Climate action, Nanotechnology, Ethylene glycol, Decentralized system, Wastewater treatment., Chemistry, Engineering, Environmental sciences, Water resources

## Abstract

Industrial wastewater containing high concentrations of ethylene glycol (EG) represents a major treatment challenge due to its high solubility, elevated chemical oxygen demand, and limited removal by conventional treatment systems. In this study, a multistage treatment strategy is proposed to overcome the demonstrated limitations of an existing industrial wastewater treatment plant for EG removal. The approach integrates solvent-assisted phase separation, coagulation–precipitation, and nanomaterial-based polishing. An external solvent-assisted phase separation step was applied as a pretreatment stage, achieving substantial reduction of the organic load (≈ 75–80% COD removal) and enabling partial recovery of an EG-rich fraction through association-driven co-extraction mechanisms rather than classical liquid–liquid extraction. Subsequent coagulation–precipitation removed suspended and colloidal matter, while tertiary polishing using nano zero-valent aluminum (nZVAl) achieved complete decoloration (100%). Kinetic analysis indicated that color removal followed Avrami-type behavior, reflecting a heterogeneous and multistep adsorption mechanism. Pilot-scale validation using real industrial wastewater confirmed the robustness of the proposed system. A preliminary techno-economic screening showed that the multistage process can operate at a net treatment cost comparable to conventional high-strength industrial wastewater treatment systems, with solvent recovery and partial EG reuse contributing to operational cost reduction rather than direct profit. Overall, the proposed framework provides a practical and scalable upgrade for industrial EG-laden wastewater treatment.

## Introduction

Industrial wastewater containing ethylene glycol (EG) is a significant environmental challenge due to its widespread use in industries such as automotive, aviation, textiles, and chemical manufacturing^1^. Ethylene glycol, a key component of antifreeze, coolants, and de-icing fluids, is highly soluble in water and exhibits a high chemical oxygen demand (COD), making it resistant to conventional wastewater treatment methods^2^. If discharged untreated, EG can contaminate aquatic ecosystems, harm biodiversity, and pose risks to human health through the contamination of drinking water sources^3^. The persistence and toxicity of ethylene glycol necessitate the development of advanced treatment strategies to ensure its effective removal from industrial effluents.

Conventional treatment methods, such as biological degradation, often struggle to completely remove ethylene glycol due to its recalcitrant nature and the presence of inhibitory by-products^4^. Biological processes, while cost-effective, are limited by the slow degradation rates of EG and the formation of toxic intermediates such as glycolaldehyde and glyoxylic acid^5^. As a result, multistage treatment approaches have gained attention for their ability to address complex wastewater matrices. These approaches typically combine physical, chemical, and biological processes to achieve higher removal efficiencies. For instance, chemical extraction can selectively recover ethylene glycol from wastewater, while coagulation/precipitation processes effectively remove suspended solids and colloidal particles^6^. Additionally, decolouration steps are often necessary to address the aesthetic and environmental concerns associated with coloured industrial effluents^7^.

Despite the widespread application of biological treatment and advanced oxidation processes (AOPs) for industrial wastewater remediation, their effectiveness for ethylene glycol (EG)-laden effluents remains limited. Biological treatment systems often exhibit slow degradation kinetics, sensitivity to shock loads, and reduced stability under high organic concentrations and fluctuating industrial conditions, particularly when dealing with highly soluble organic compounds such as EG^2,8^. In parallel, AOPs such as Fenton and Fenton-like processes can achieve partial oxidation of organic contaminants; however, their performance is frequently constrained by high chemical consumption, radical scavenging in complex wastewater matrices, and the generation of iron-rich secondary sludge^9,10^.

Moreover, most existing treatment strategies are primarily designed for pollutant degradation rather than resource recovery, thereby overlooking the potential reuse of EG as a valuable industrial chemical^11,12^. This creates a clear research gap in developing treatment approaches that can simultaneously address the complex organic composition of EG-containing industrial wastewater, reduce the overall pollutant load, and enable resource-oriented recovery with practical scalability^13^.

The integration of chemical extraction, coagulation/precipitation, and decolouration processes in a multistage treatment system offers a promising solution for the effective remediation of ethylene glycol-laden wastewater. Chemical extraction, often using solvents or adsorbents, can recover valuable ethylene glycol for reuse, reducing resource consumption and promoting circular economy principles^14^. Coagulation/precipitation, using coagulants such as ferric chloride or aluminum sulfate, targets the removal of suspended solids, organic matter, and phosphates, enhancing the overall treatment efficiency. Decolouration, achieved through advanced oxidation processes (AOPs) or adsorption, ensures the aesthetic quality of the treated water, making it suitable for discharge or reuse^15,16^.

The development of such a multistage treatment system is critical for industries generating ethylene glycol-laden wastewater, as it aligns with global efforts to promote sustainable industrial practices and comply with stringent environmental regulations^17^. For example, the European Union’s Water Framework Directive and the United States Environmental Protection Agency (EPA) have set strict limits on the discharge of organic pollutants, including ethylene glycol, into water bodies^18^. By combining these processes, the proposed system aims to achieve high removal efficiency for ethylene glycol, COD, and colour, while minimizing environmental impact and operational costs.

Nanotechnology, particularly the use of zero-valent metals, has gained significant attention for the removal of organic and inorganic contaminants from wastewater, owing to their high reactivity and extensive surface area. Numerous studies have explored the application of both magnetic and nonmagnetic zero-valent metals in wastewater treatment. These processes primarily target the removal of organic pollutants, including colour, Polycyclic Aromatic Hydrocarbons (PAHs), BTEX compounds, Chemical Oxygen Demand (COD), and Total Organic Carbon (TOC), through adsorption and degradation mechanisms using nano zero-valent iron and its derivatives^19–22^. Additionally, nanomagnetic sorbents have demonstrated effectiveness in eliminating various inorganic contaminants^23–26^. For instance, Mahmoud et al. (2019) investigated the adsorption behavior of chemically synthesized nZVAl for the removal of soluble organic matter from municipal wastewater. Their findings highlighted the capability of nonmagnetic nanoparticles to adsorb a broad spectrum of soluble organic pollutants from aqueous solutions^21^.

This study not only addresses the technical challenges of ethylene glycol removal but also contributes to the broader goal of achieving zero liquid discharge (ZLD) in industrial wastewater management^27^. The findings of this research are expected to provide valuable insights into the design and optimization of multistage treatment systems for complex industrial effluents. Furthermore, the integration of resource recovery (e.g., ethylene glycol reuse) and advanced treatment technologies represents a step forward in sustainable wastewater management, aligning with the principles of the circular economy^28^.

The novelty of this work lies in designing a treatment - recovery framework rather than applying isolated unit operations. The study integrates (i) a solvent-assisted phase-separation step that enables partial recovery of ethylene glycol (EG) together with a substantial reduction of COD-associated organic load, (ii) an optimized coagulation/precipitation stage for suspended and colloidal matter removal, and (iii) a tertiary polishing unit employing nano zero-valent aluminum (nZVAl) based media for complete decolouration. Unlike conventional studies that evaluate coagulation or nanomaterials as standalone options, the present work provides a staged performance map, discusses operational trade-offs (chemical demand, sludge formation, solvent recycling), and links pollutant removal to a practical resource recovery pathway.

## Materials and procedures

### Synthesis and storage of aluminum nanoparticles

Aluminum nanoparticles were synthesized via a chemical reduction route. Briefly, 13.69 g of Al₂(SO₄)₃·18 H₂O was dissolved in a mixed solvent consisting of 50 mL deionized water (DW) and 50 mL ethanol. Separately, 9.1 g of NaBH₄ was dissolved in 1.0 L of DW to prepare the reducing agent solution. The NaBH₄ solution was subsequently transferred to a burette and added dropwise to the aluminum precursor solution at a controlled rate of 0.1 mL every 4 s under continuous stirring. The formation of a white precipitate was observed almost immediately following the addition of the first drops of the reducing agent, indicating rapid reduction of aluminum species.

Following completion of the reduction reaction, the resulting nano zero-valent aluminum (nZVAl) particles were separated and thoroughly washed with deionized water and ethanol to remove residual salts and reaction by-products. The washed nanoparticles were then dried under controlled conditions. Throughout post-synthesis handling, particular care was taken to minimize exposure to atmospheric oxygen and moisture.

To preserve the reactivity of the synthesized nZVAl and prevent oxidative degradation, the dried powder was immediately transferred into airtight storage containers under an inert nitrogen atmosphere. The containers were purged with high-purity nitrogen gas and tightly sealed to maintain oxygen-free conditions. Under these storage conditions, the nanoparticles retained their metallic appearance and functional reactivity throughout the experimental period, with no visible signs of oxidation or agglomeration prior to use.

Although aluminum nanoparticles are inherently susceptible to surface oxidation, the formation of a thin and stable oxide layer is well recognized to act as a passivation barrier, thereby limiting further oxidation of the metallic core. In the present study, the controlled inert storage environment was sufficient to maintain the functional performance of nZVAl within the timeframe of experimental application. All synthesized materials were utilized within a defined storage period to ensure consistent behavior during batch and pilot-scale experiments.

### Batch studies

In the initial batch experiments, dichloromethane was employed for the solvent-assisted phase separation of ethylene glycol–associated organic fractions from the industrial wastewater. Following the extraction step, preliminary coagulation screening tests were conducted to evaluate the coagulation behavior of the post-extraction wastewater. The samples were mixed with selected coagulants, including polyacrylamide, ferric chloride, and aluminum sulfate, using a standard jar test apparatus.

Rapid mixing was applied at 150 rpm for 3 min to ensure uniform dispersion of the coagulants, followed by slow agitation at 30 rpm for 20 min to promote floc formation. After complete precipitation and settling, the coagulation performance was comparatively evaluated based on floc formation efficiency, settling behavior, and clarity of the supernatant. The most effective coagulant was subsequently identified for further assessment.

The adsorption and degradation processes of colour using nano zero-valent aluminum (nZVAl) were studied through a batch technique, focusing on a single variable at a time. The operating conditions, including pH, adsorbent dose, contact time, and stirring rate, were optimized based on previous studies and minimum effective results^29^.

Table 1 summarizes the batch experiments conducted under various operating conditions. A known weight of nano aluminum (0.15 g) was equilibrated with 1000 mL of partially treated wastewater in Erlenmeyer flasks and shaken for a specified period at room temperature. After equilibration, the adsorbent suspension was separated using Whatman filter paper No. 2, and the remaining concentrations of COD, BOD, TSS, TN, and TP were measured according to the Standard Methods for the Examination of Water and Wastewater, 23rd edition^30^. The colour removal efficiency was calculated using Eq. (1)^30^. The amount of sorbed colour was calculated using Eq. (2)^22,31^. All experiments were performed in triplicate, and all results were within lab measurements.

1 $$\:Sorption\:\left(\%\right)=\left(\:\frac{{C}_{0}-{C}_{e}}{{C}_{0}}\:\right)\times\:100$$

where C_o_ is the initial concentration (mg/L) and C_e_ is the equilibrium concentration in solution (mg/L).

2 $$\:{Q}_{e}\left(mg/g\right)=\frac{\left({C}_{0}-{C}_{e}\right)V}{m}$$

where q_e_ is the equilibrium adsorption capacity (mg/g), V is the volume of aqueous solution (L), and m is the dry weight of the adsorbent (mg).

**Table 1:** Batch experiments at different operating parameters.

Effect*	Dose (g)	Contact time (min)	pH	Stirring rate (rpm)	Temperature (°C)
polyacrylamide, *ferric chloride*, and aluminum sulfate					
Effect of pH	0.75	3 mixing/20 flocs formation/30 settling	4–10	150 and 30	25
Effect of dose	0.25–1.25	3 mixing/20 flocs formation/30 settling	8	150 and 30	25
nZVAl					
Effect of pH	0.7	40	6–9	100	25
Effect of adsorbent dose	0.05–0.25	40	7.5	100	25
Effect of contact time	0.7	5–60	7.5	100	25
Effect of stirring rate	0.7	40	7.5	100–500	25

* All experiments were performed in triplicate at room temperature (25 ± 1 °C).

### Kinetic studies

Kinetic experiments were conducted to determine the contact time required to reach adsorption equilibrium and to elucidate the governing removal mechanisms. Standard aqueous solutions were brought into contact with the adsorbent at predetermined time intervals under constant temperature and controlled experimental conditions. The amount of adsorbed contaminant at any time t, denoted as $\:{q}_{t}$ (mg g⁻¹), was calculated according to Eq. (3):

3 $$\:{Q}_{t}=\frac{\left({C}_{o}-{C}_{t}\right)V}{W}$$

where $\:{C}_{0}$(mg L^−1^) is the initial colour concentration, $\:{C}_{t}$(mg L^−1^) is the colour concentration at time t, V (L) is the solution volume, and W (g) is the mass of the nZVAl.

To identify the most appropriate kinetic model describing the adsorption process at different contact times, several nonlinear kinetic models were evaluated, including the pseudo-first-order, pseudo-second-order, Elovich, Avrami, and intraparticle diffusion models. These models were selected to distinguish between physisorption, chemisorption, surface heterogeneity effects, and mass transfer limitations^32–36^. A summary of the theoretical assumptions and governing equations of each kinetic model is presented in Table 2.

**Table 2:** Nonlinear kinetic models applied to describe colour adsorption- degradation behavior.

Kinetic model name	Description	Equations	Equation No.
Pseudo First Order	Assumes adsorption governed primarily by weak physical interactions such as van der Waals forces and hydrogen bonding, indicative of physisorption.	$\:{Q}_{t}=\:{Q}_{e}(1-{e}^{(-{K}_{1}t})$	Eq. (4)
Pseudo Second Order	Assumes adsorption occurs through electron sharing or exchange between adsorbent and adsorbate, characteristic of chemisorption.	$\:{Q}_{t}=\:\frac{{Q}_{e}^{2}{K}_{2}t}{(1+{Q}_{e}{K}_{2}t)}$	Eq. (5)
Elovich	Commonly applied to heterogeneous solid surfaces and chemisorption-controlled processes; suitable for describing adsorption from aqueous solutions onto energetically non-uniform surfaces.	$\:{Q}_{t}=\:\frac{1}{\beta\:}Ln\left(\alpha\:{\beta\:}_{t}\right)$	Eq. (6)
Avrami	Originally developed for crystallization kinetics; applied here to account for complex adsorption mechanisms involving phase transformation and variable reaction rates.	$\:{Q}_{t}=\:{Q}_{e}(1-{e}^{(-{K}_{av}{t}^{{n}_{av}}})$	Eq. (7)
Intraparticle model	Describes diffusion-controlled adsorption where solute transport within the adsorbent pores governs the rate; film diffusion resistance is neglected.	$\:{Q}_{t}=\:{K}_{id}{t}^{0.5}+{C}_{i}$	Eq, (8)

To quantitatively evaluate the fitness of each kinetic model, nonlinear regression analysis was performed using an error function approach. Marquardt’s Percent Standard Deviation (MPSD) was employed as the primary statistical criterion to assess model adequacy and minimize deviations between experimentally measured and calculated adsorption capacities. The MPSD was calculated using Eq. (4):

9 $$\:\sum\:_{i=1}^{p}{\left(\frac{{q}_{e,\:meas}-{q}_{e,\:calc}}{{q}_{e,\:meas}}\right)}_{i}^{2}$$

where $\:{q}_{e,\mathrm{meas}}$ and $\:{q}_{e,\mathrm{calc}}$ represent the experimentally measured and model-predicted equilibrium adsorption capacities, respectively, and p is the number of experimental data points.

Based on the lowest MPSD values and the best agreement between experimental and predicted data, the selected nonlinear kinetic model was identified as the most suitable descriptor of the adsorption mechanism, consistent with previous findings reported by^37^.

### Industrial wastewater source and site description

**Fig. 1:**
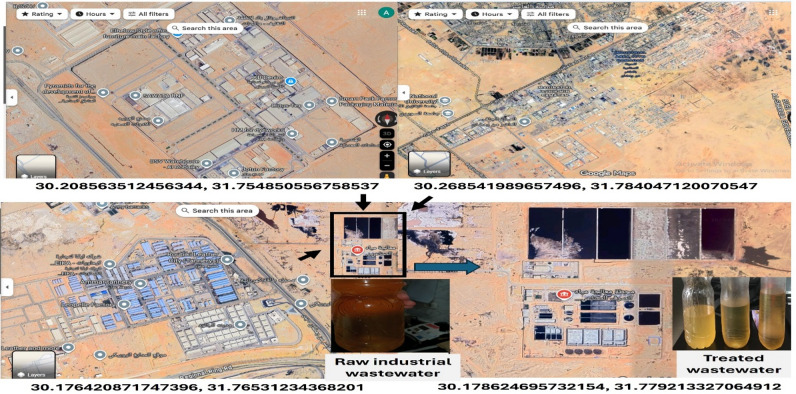
Satellite imagery of the industrial complex and centralized industrial wastewater treatment facility from which samples were collected, along with photographs of the raw and treated industrial wastewater.

The wastewater samples used in this study were collected from a centralized industrial wastewater treatment facility serving a large manufacturing complex located in a desert-based industrial zone (Fig. 1). The satellite imagery of the area shows an extensive industrial cluster composed of mixed-type factories, including metalworking workshops, chemical processing units, mechanical assembly facilities, storage yards, and light manufacturing plants. These industrial activities discharge their liquid effluents into a shared industrial sewer network that ultimately flows to a centralized treatment station equipped with sedimentation tanks, equalization basins, and multiple evaporation ponds. The nature of the industrial estate implies a diverse wastewater composition, typically containing mixtures of ethylene glycol (EG) from cooling and heat-transfer systems, organic pollutants, lubricating oils, detergents and surfactants, suspended solids, high-salinity streams, and variable loads of COD and BOD. Table 3 shows the characteristics of industrial wastewater before and after existing industrial WWTP treatment (traditional). The presence of evaporation ponds and sludge-handling areas further indicates that the facility receives high-strength industrial effluents requiring physical–chemical pretreatment prior to biological or polishing stages. Such complex wastewater characteristics suggest that EG in the collected samples is not present as a free, isolated compound but rather exists partially associated with organic matrices, surfactant-rich phases, oily clusters, or colloidal aggregates produced by the combination of industrial discharges. This matrix complexity is consistent with the observed behavior during the DCM-assisted separation step, where solvent partitioning affected not only dissolved EG but also EG-bearing organic assemblies. The compositional nature of the sampling site therefore provides a realistic context for the co-extraction and phase-association mechanisms described in this study.

**Table 3:** Characteristics of industrial wastewater before and after existing centralized industrial WWTP.

Parameter	Unit	Raw	Treated	SD ±	Limits: law 93/1962^*^
pH	--	5.52 ± 0.2	6.9	0.2	6–9.5.5
Chemical oxygen demand (COD)	mg/L	8650 ± 98	1960	34	1100
Soluble -COD		7860 ± 86	1830	22	-
Biological oxygen demand (BOD)		1970 ± 35	996	32	600
Total suspended solids (TSS)		2037 ± 53	1047	21	800
Total phosphorus (TP)		8.6 ± 0.4	6.8	0.3	25
Total nitrogen (TN)		74.2 ± 3.7	60.4	3.0	100
Oil		6.93 ± 0.35	4.6	0.23	100
Hydrogen sulfide (H_2_S)		48.9 ± 2.5	16.8	1.0	10
Ethylene glycol		3700 ± 77	607	26	-
CN		0.025 ± 0.001	< 0.001	0	0.2
Phenols		< 0.00001	< 0.00001	0	0.05
Cadmium (Cd)		0.003 ± 0.001	< 0.00001	0	0.2
Lead (Pb)		0.001 ± 0.001	< 0.00001	0	1
Copper (Cu)		0.009 ± 0.001	< 0.00001	0	1.5
Aluminum (Al)		0.092 ± 0.009	0.055	0.001	1.0
Nickel (Ni)		0.056 ± 0.005	< 0.00001	0	1
Arsenic (As)		0.027 ± 0.001	< 0.00001	0	2
Chromium (Cr)		4.80 ± 0.48	2.26	0.11	0.2
Mercury (Hg)		< 0.00001	< 0.00001	0	0.2
Tin (Sn)		< 0.00001	< 0.00001	0	2
Boron (B)		0.01 ± 0.001	< 0.00001	0	1
Colour	Pt/co	683 ± 23	386	18	---
Deposits after 10 min	cm^3^	8	3	-	8
Deposits after 30 min	cm^3^	12	7	-	15

^*^Limit 93/1962: discharge of wastewater to public sewers.

### Quality assurance and safety compliance

All analytical measurements reported in this study were conducted in an accredited laboratory operating in accordance with ISO/IEC 17,025, ensuring the reliability, traceability, and quality control of the generated data. Instrument calibration, method validation, and quality assurance procedures were performed following standardized protocols within the accredited scope of the laboratory.

Field-scale and pilot-scale experiments were carried out under an environmental and occupational safety management framework compliant with ISO 14,001 and ISO 45,001, respectively. These standards ensured that all experimental activities were conducted with appropriate environmental controls, risk assessments, and occupational health and safety measures. The integration of accredited analytical practices with certified environmental and safety management systems further supports the robustness, reproducibility, and regulatory relevance of the experimental results presented in this study.

### Sludge generation and disposal

Sludge generation during coagulation and phase separation experiments was evaluated using standard settling tests. The settled sludge volume was recorded after 10 and 30 min of quiescent settling and is reported in Table 3. The resulting sludge consisted mainly of coagulated organic matter and inorganic precipitates. Following characterization, the sludge was collected and dewatered, then disposed of in accordance with applicable local environmental regulations for industrial wastewater treatment residues. Due to the pilot-scale nature of the study, sludge volumes were limited and did not pose operational constraints.

## Results and discussion

### Characterization of nZVAl

**Fig. 2:**
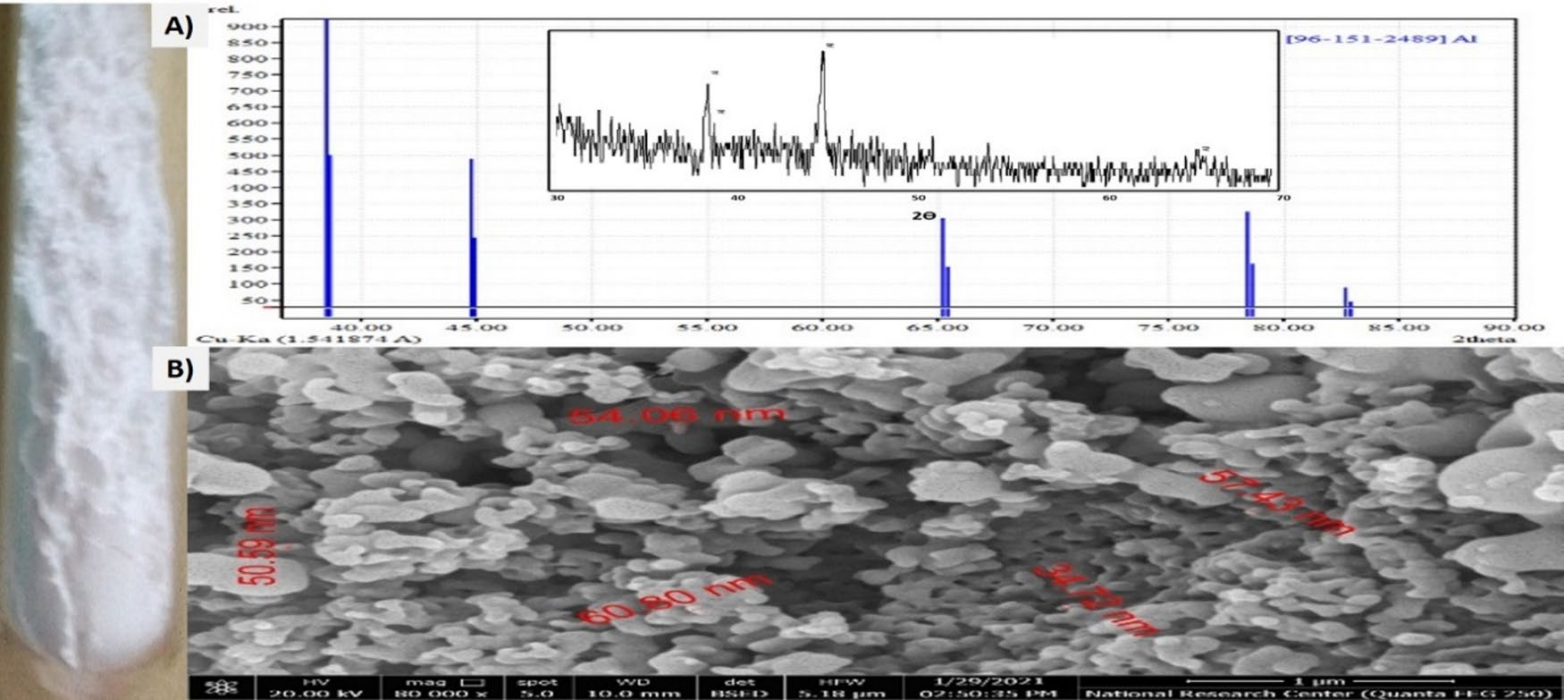
**a**) XRD of powder nZVAl, and **b**) SEM of the prepared nZVAl.

**Fig. 3:**
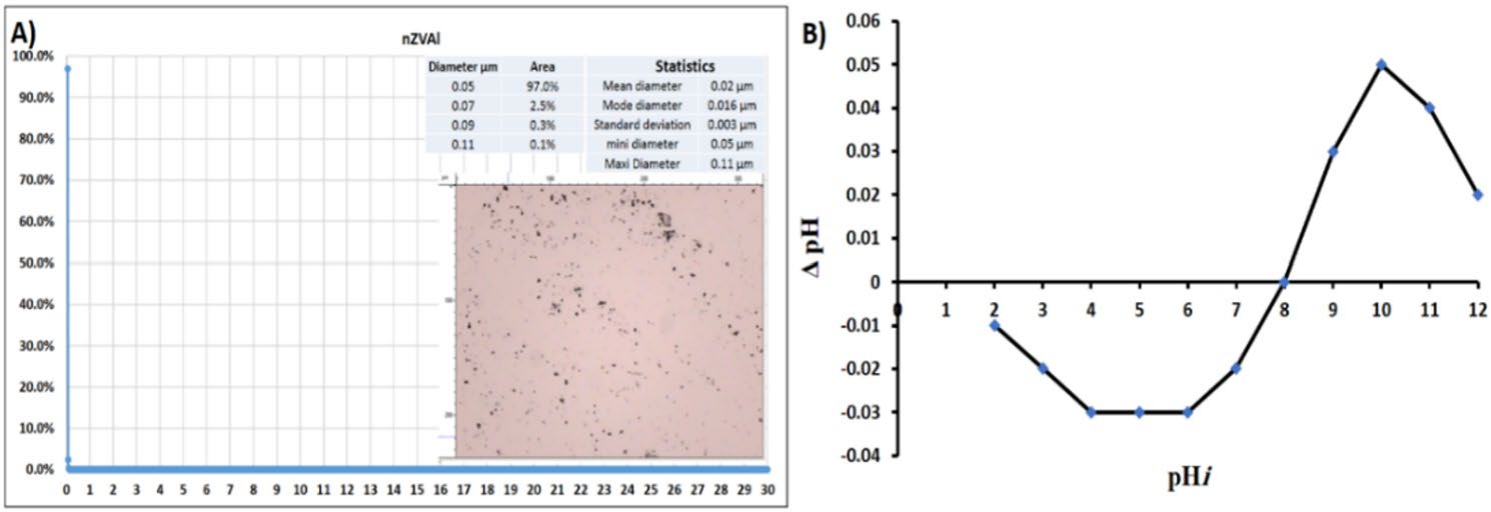
**a**) Particle size distribution of nZVAl in bare powder, and **b**) PZC of the prepared nZVAl.

The XRD patterns for powder nano aluminum with an angle (2Ө) ranging from 35° to 90° are illustrated in Fig. 2a. With five detected peaks at 2Ө (= 38.57°, 44.95°, 65.27°, 78.68°, and 82.92°, the XRD pattern demonstrated the synthesis of pure referenced aluminum [96–151−2489]. An SEM picture of the synthetic nZVAl may be seen in Fig. 2b. The findings showed the development of a crystal size of 60 nm for powder aluminum, as well as the existence of an uneven surface structure^38^. As shown in Fig. 3a, the particle-size-distribution of nZVAl in bare powder was evaluated from 0 to 30 μm at a rate of 0.02 mm. The findings showed that 97% of the nZVAl size was 50 nm. Figure 3b shows the synthesized nZVAl powder’s PZC. According to the obtained results, the nZVAl_PZC_ was around 8.0, showing agreement with the previous results^38^.

### Treatment process

The proposed multistage treatment system for industrial ethylene glycol (EG)-laden wastewater demonstrates significant advancements in both environmental remediation and cost efficiency. The integration of chemical extraction, coagulation/precipitation, and decolouration processes offers a robust solution for the treatment of complex industrial effluents, aligning with global sustainability goals and stringent environmental regulations.

#### Solvent-assisted phase separation

**Fig. 4:**
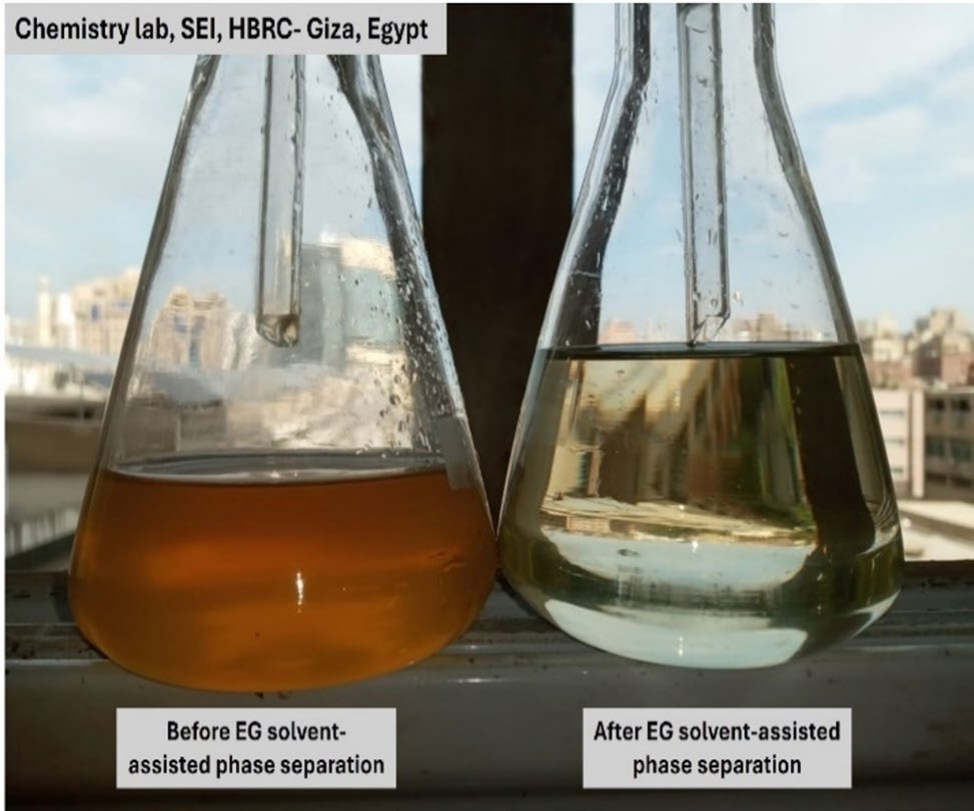
Solvent-assisted phase separation.

The use of dichloromethane (DCM) as a solvent-assisted phase separation step resulted in substantial reductions in key pollutants as shown in Figure 4, including 78.8% COD, 77.7% BOD, 71.9% TSS, 61.8% sulfides, and 63.2% colour. The successful recovery of a measurable EG-rich fraction, combined with high DCM recyclability using rotary evaporation, demonstrates that the solvent step provides both treatment and resource-recovery benefits while maintaining a low environmental footprint^3^. It is essential to clarify that DCM does not extract free ethylene glycol (EG) through classical liquid–liquid equilibrium, given the complete water miscibility of EG. Instead, the observed extraction behavior is better explained by co-extraction and association-driven mechanisms. Previous analytical studies have shown that DCM can separate poly (ethylene glycol) (PEG) fractions from aqueous matrices despite their high-water solubility, due to solvent-induced disruption of organic clusters and migration of associated organics into the organic phase (PEG fractionation study). Such precedents support the hypothesis that in industrial EG effluents—where EG is partially associated with COD-rich organic matter, surfactants, micelles, and colloidal structures—DCM does not selectively extract EG molecules but rather extracts the organic-associated EG fraction as part of a complex separable organic bundle. This mechanism provides a scientifically coherent explanation for the experimentally observed EG recovery and the significant reduction in organic load achieved during the solvent-assisted phase separation step^39^.

The selection of dichloromethane (DCM) was based on process-oriented considerations rather than conventional solvent extraction of free ethylene glycol. Although greener solvents and membrane-based separation technologies are increasingly explored for wastewater treatment, their applicability to EG-laden industrial effluents remains limited under highly complex conditions. In the present study, DCM was favored due to its immiscibility with water, high volatility enabling efficient solvent recovery, and its demonstrated ability to disrupt EG-associated organic clusters. Membrane-based approaches were considered less suitable owing to fouling susceptibility and reduced performance in the presence of surfactants and suspended solids. These considerations justified the use of DCM at the pilot scale, while acknowledging that future work may investigate greener solvent alternatives or hybrid separation systems (Fig. 4).

#### Secondary treatment (selecting coagulant)

**Fig. 5:**
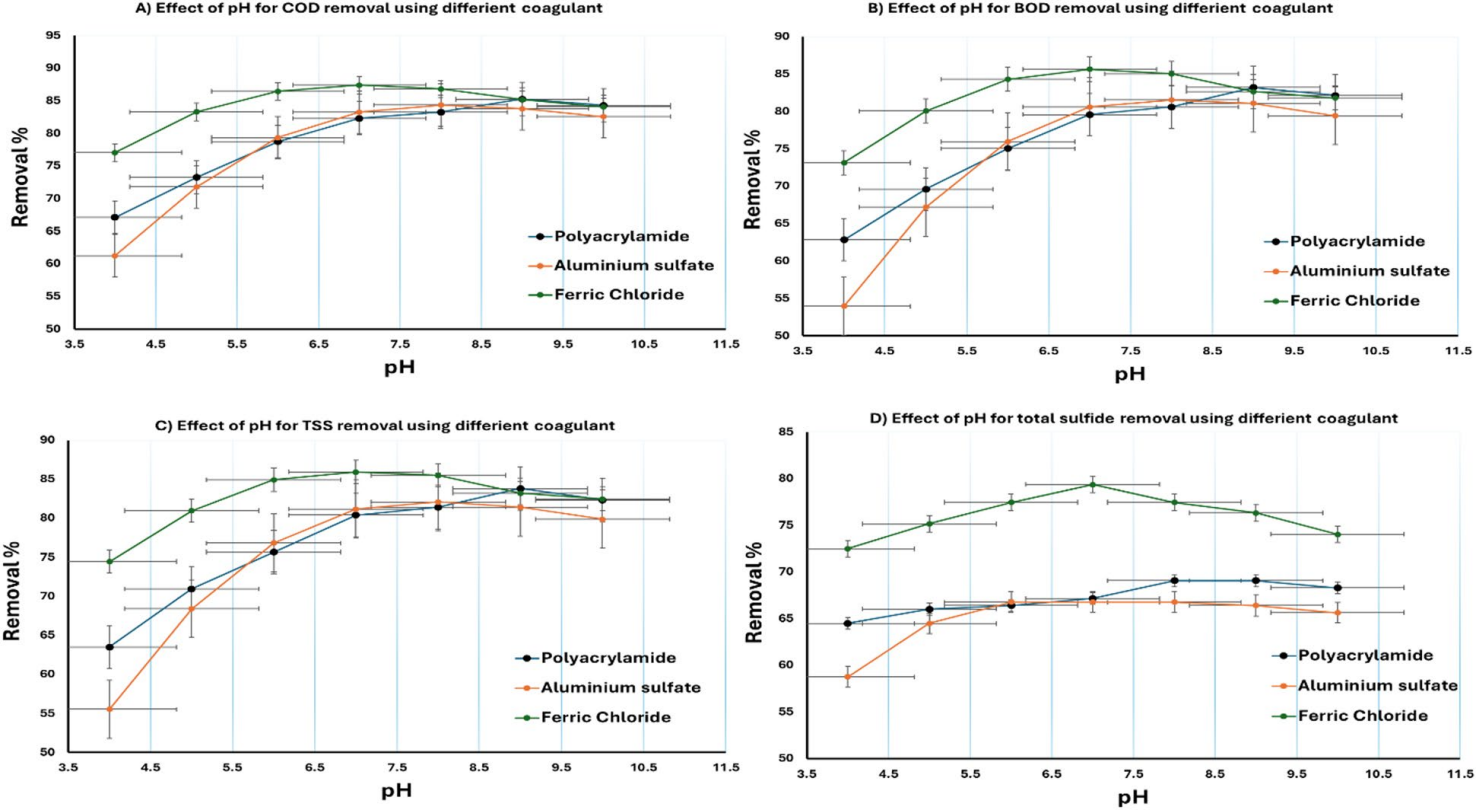
Effect of pH on the COD, BOD, TSS, and sulfide removal efficiency.

The most effective coagulant was selected according to the most effective removal efficiency at different pH. Figure 5 describes the removal efficiency of wastewater contaminants at different pH levels. The results indicated that the Ferric chloride is the most effective coagulant after separation technique.

**Fig. 6:**
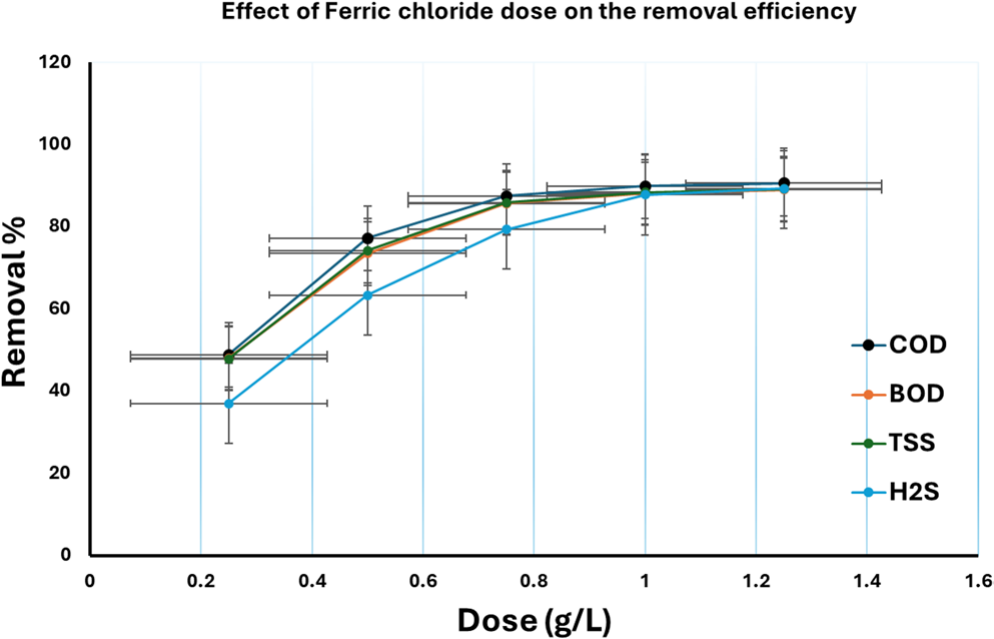
Effect of ferric chloride dose on COD, BOD, TSS, and sulfide removal efficiency.

After coagulant selection. The effect of ferric chloride dose was studied using different doses as shown in Fig. 6. The results obtained indicated that the minimum effective dose was 0.75 g/L. The coagulation process is particularly effective in removing suspended solids and colloidal particles, which are often challenging to treat using conventional biological methods^6^.

#### Decolouration with nano Zero-Valent aluminum (nZVAl)

Nano zero-valent aluminum (nZVAl) was selected as the tertiary polishing medium in this study due to its strong affinity toward organic matter and colour-causing compounds through adsorption-dominated surface interactions. Unlike nano zero-valent iron (nZVI), which primarily promotes reductive transformations and is often associated with rapid surface passivation, secondary iron release, and increased sludge generation under high organic loading, nZVAl provides a more suitable mechanism for post-treatment polishing applications^40–42^.

Aluminum-based materials are widely used in conventional water and wastewater treatment for organic matter destabilization and coagulation, and their nanoscale counterparts exhibit enhanced surface reactivity and adsorption capacity^42–44^. In the present multistage treatment configuration, nZVAl was therefore employed specifically to enhance decolouration and removal of residual soluble organics following upstream separation and batch treatment stages, rather than as a standalone remediation agent. This functional distinction justified the selection of nZVAl over iron-based or other metallic nanoparticles within the proposed treatment framework.

**Fig. 7:**
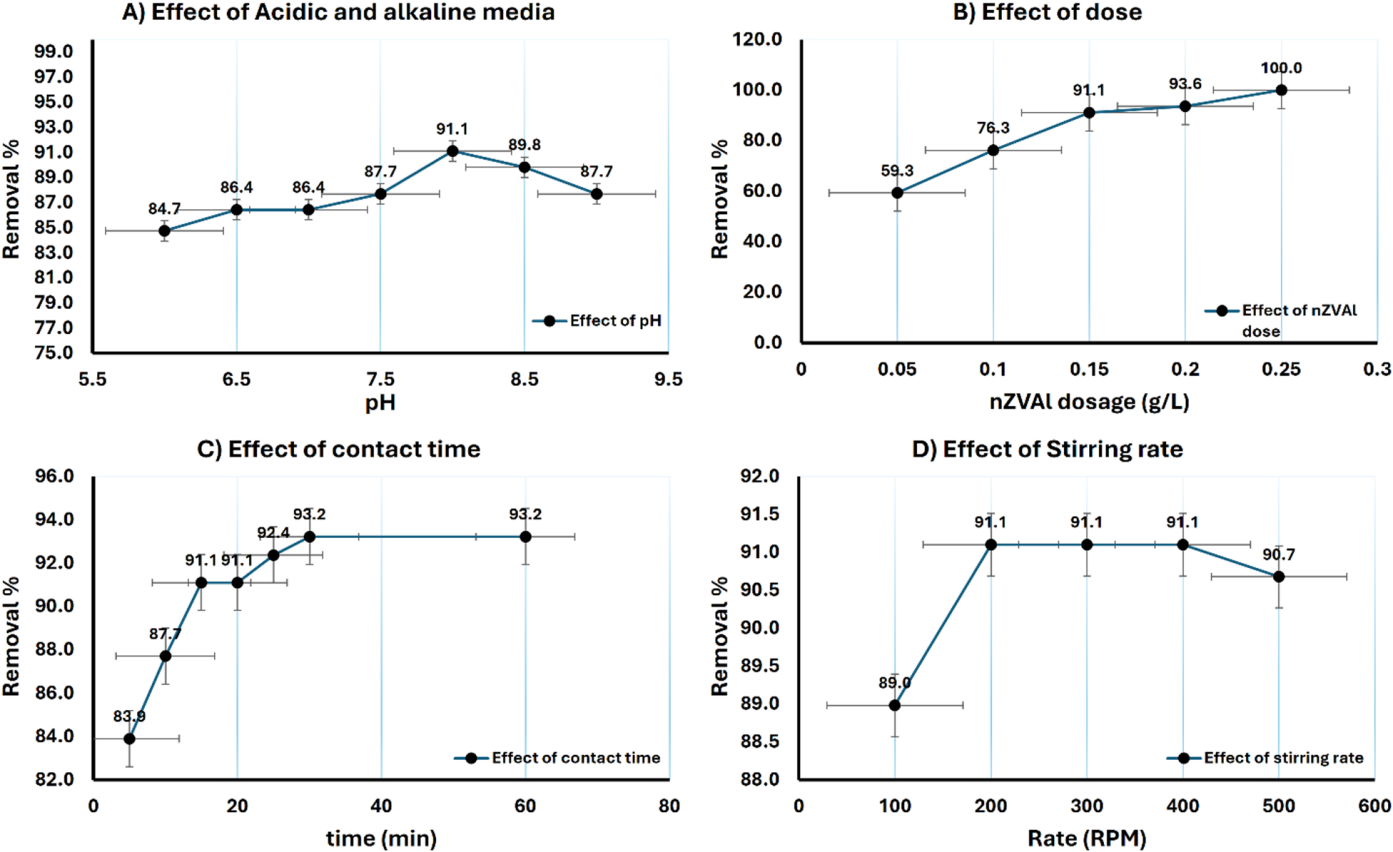
Effect of operating conditions for colour degradation using nZVAl.

The effect of nZVAl was studied for colour removal and degradation as tertiary treatment step and the obtained results shown in Fig. 7. The effect of operating conditions (pH, dose, contact time and stirring rate was studied for best removal treatment and the obtained results indicated that about 91.1% of colour removal was observed using 0.15 g of powder nZVAl at pH 8 (at zero charge), and stirring rate 100 rpm for 15 min. The final polishing step using a filtration media composed of collected nano zero-valent aluminum (nZVAl), birm, and activated carbon achieved 100% colour removal. The use of nZVAl for decolouration is supported by previous studies that demonstrate the high reactivity and extensive surface area of zero-valent metals in adsorbing and degrading organic pollutants, including colourants^45^. The integration of nanotechnology in wastewater treatment has shown promising results in enhancing the removal of persistent organic pollutants, making it a valuable addition to multistage treatment systems^46^.

##### Kinetic studies

**Fig. 8:**
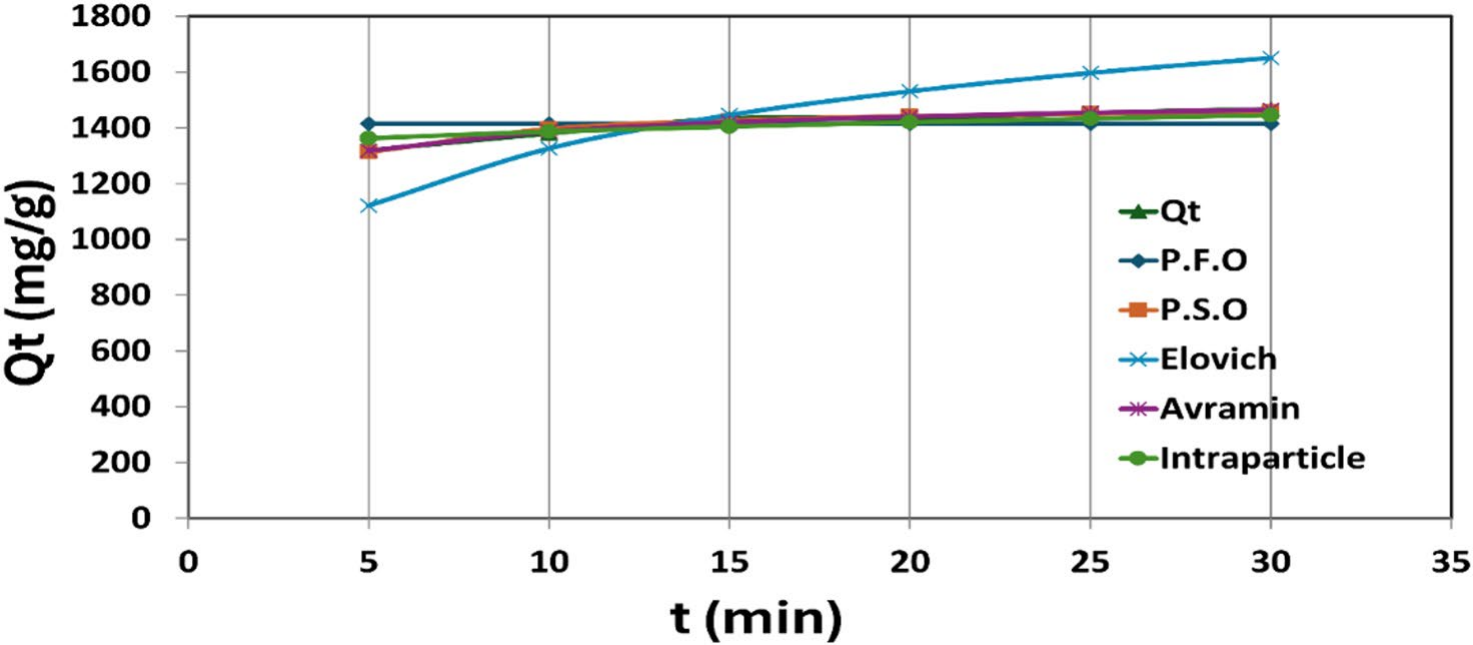
nonlinear kinetic studies for colour removal using nZVAl.

Kinetic studies were conducted to elucidate the adsorption behavior and removal mechanism of colour onto nano zero-valent aluminum (nZVAl) as shown in Fig. 8. The experiments were designed to evaluate the relationship between adsorption capacity and contact time under controlled conditions, considering the variation between initial and residual colour concentrations. Contact times ranging from 5 to 30 min were investigated, while the 60 min interval was excluded from kinetic modeling due to the absence of significant changes in colour removal beyond equilibrium, indicating saturation of active adsorption sites.

The amount of colour adsorbed at any given time (qₜ) was calculated and correlated with contact time to assess the adsorption rate and governing mechanism. Several nonlinear kinetic models were applied, including pseudo-first order (PFO), pseudo-second-order (PSO), Elovich, Avrami, and intraparticle diffusion models. Model performance was evaluated using the Marquardt’s Percent Standard Deviation (MPSD) error function as a statistical criterion for goodness of fit.

The kinetic results demonstrated that the Avrami model provided the best description of the adsorption process, exhibiting the lowest MPSD value (0.00014) among all tested models. This finding suggests that the adsorption mechanism involves a complex process governed by heterogeneous surface interactions and variable adsorption rates rather than a single-step physical or chemical adsorption mechanism. The pseudo-second-order model showed the second-best fit (MPSD = 0.00026), indicating a partial contribution of chemisorption mechanisms. In contrast, higher MPSD values obtained for the pseudo-first-order, Elovich, and intraparticle diffusion models indicate less satisfactory representation of the experimental data.

The dominance of the Avrami kinetic model highlights the role of surface heterogeneity and time-dependent adsorption behavior in the interaction between colour-causing compounds and nZVAl particles. These results confirm that the adsorption process is not governed solely by intraparticle diffusion or simple physisorption but involves a multistep adsorption mechanism.

**Table 4.** Kinetic parameters and nonlinear model fitting results for colour adsorption onto nZVAl.

Model	Constants		MPSD error
Pseudo-first order	Q_e_	1414.444	0.00807
	K_1_	7.444	
Pseudo-second order	Q_e_	1490.381	0.00026
	K_2_	0.001	
Elovich	alpha	2623.742	0.05442
	beta	0.003	
Avrami	Q_e_	1531.958	0.00014
	K_av_	1.303	
	n_av_	0.258	
Intraparticle diffusion	K_id_	25.45	0.00198
	Ci	1305.683	

The kinetic parameters summarized in Table 4. reveal a strong consistency between experimental and model-predicted adsorption capacities. Among the tested models, the Avrami kinetic model exhibited the lowest Marquardt’s Percent Standard Deviation (MPSD = 0.00014), indicating superior agreement with the experimental data compared to pseudo-first-order, pseudo-second-order, Elovich, and intraparticle diffusion models.

The Avrami rate constant ($\:{k}_{\mathrm{av}}=1.303$) reflects a relatively rapid adsorption process, while the Avrami exponent ($\:{n}_{\mathrm{av}}=0.258$) provides critical insight into the adsorption mechanism. The value of $\:{n}_{\mathrm{av}}<1$ suggests a heterogeneous adsorption process governed by multiple adsorption sites with varying energy levels and time-dependent surface interactions. Such behavior is characteristic of complex adsorption systems involving surface restructuring, gradual site occupation, and non-uniform adsorbate–adsorbent interactions rather than a single-rate limiting step.

The second-best fit obtained using the pseudo-second-order model (MPSD = 0.00026) indicates that chemisorption contributes partially to the overall removal mechanism. However, the superior performance of the Avrami model confirms that colour removal by nZVAl proceeds through a multistep adsorption mechanism rather than simple physisorption or intraparticle diffusion alone. These findings are consistent with previous studies reporting Avrami-controlled kinetics for adsorption processes on heterogeneous nanostructured surfaces^47^.

#### Application in real wastewater treatment pilot system

**Fig. 9:**
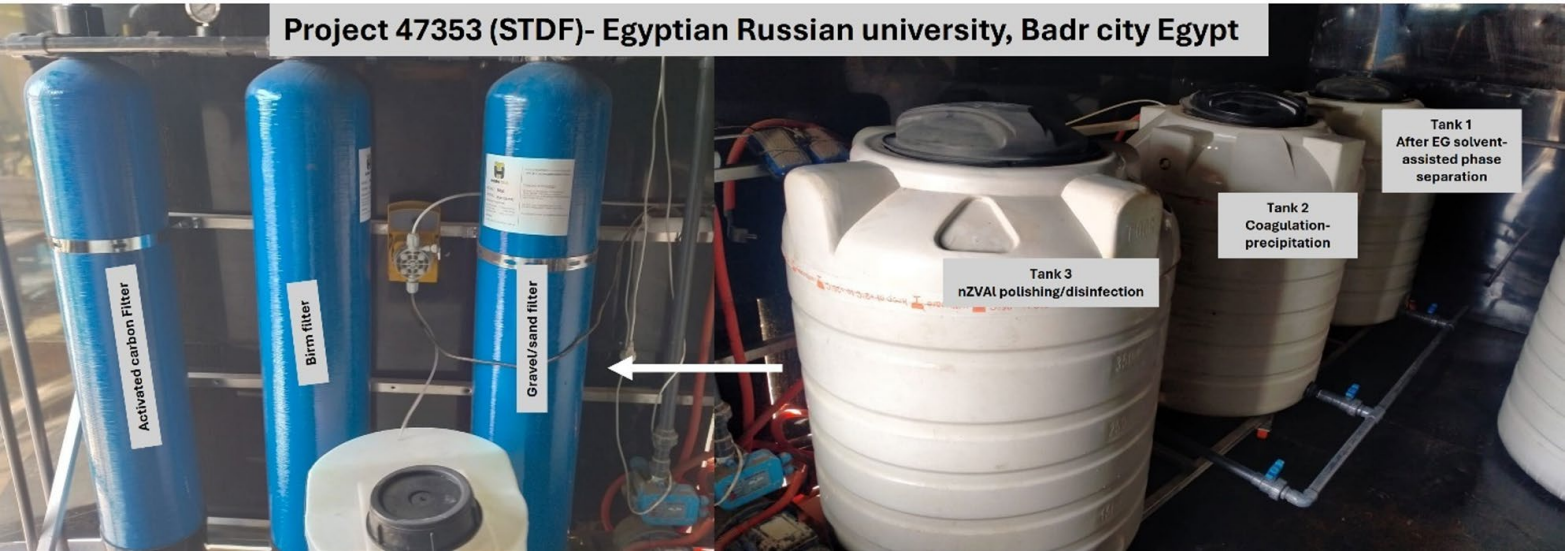
Pilot multistage treatment system.

A pilot-scale wastewater treatment system was constructed and operated to evaluate the practical applicability of the proposed multistage treatment approach under real industrial conditions. The pilot system was developed as part of Project 47,353 (STDF) at the Egyptian Russian University, Badr City, Egypt. As shown in Fig. 9, the system consists of three interconnected treatment tanks, supported by granular activated carbon and gravel filtration units, designed to validate the efficiency and scalability of the process. Prior to feeding the pilot system, raw industrial wastewater underwent external solvent-assisted phase separation using DCM to remove a significant portion of the EG-associated organic fraction. Accordingly, Tank 1 received the partially clarified wastewater after external EG separation, functioning as the equalization and feed reservoir for subsequent treatment steps. From Tank 1, the wastewater was transferred to Tank 2, where the physical–chemical treatment phase took place, including settling, filtration, and controlled recirculation. Final polishing was performed in Tank 3, which contained a packed bed of nano zero-valent aluminum (nZVAl), providing complete decolouration and additional removal of residual organics.

In parallel with the solvent-based approach, advanced oxidation experiments using a Fenton-like system (Fe²⁺/H₂O₂) were performed to assess their potential as an alternative pretreatment method. Although the oxidation process achieved partial degradation of organic pollutants, its removal efficiencies for COD, BOD, and colour were considerably lower than those achieved by the solvent-assisted phase separation. The reduced performance of the Fenton-like system is attributed to the complex nature of the industrial wastewater, which contains high levels of surfactants, oils, salts, and EG-associated organic clusters known to quench hydroxyl radicals and impede oxidation efficiency. Furthermore, the process produced substantial iron-rich sludge, increasing chemical demand and handling requirements.

In contrast, the solvent-assisted phase separation demonstrated superior performance, achieving substantial pollutant reduction while enabling resource recovery through EG separation and minimizing secondary waste generation. The successful operation of the pilot system confirmed that the proposed multistage treatment—when preceded by external EG phase separation—offers a robust, scalable, and industrially feasible solution for treating complex EG-laden wastewater streams.

**Table 5:** Characteristics of industrial wastewater before and after current multistage treatment system.

Parameter	Unit	Raw	After solvent-assisted phase separation	After coagulation/precipitation	After nZVAl	After filtration (Gravel/Sand- Birm- Activated carbon)	After microfiltration	Limits: law 93/1962^*^
pH	--	5.52 ± 0.2	6.25 ± 0.2	6.58 ± 0.2	6.93 ± 0.2	6.88 ± 0.2	6.73 ± 0.2	6–9.5.5
Chemical oxygen demand (COD)	mg/L	8650 ± 98	3570 ± 52	650 ± 23	93 ± 12	85 ± 12	85 ± 12	1100
Soluble -COD		7860 ± 86	3127 ± 39	593 ± 18	82 ± 10	81 ± 10	80 ± 10	-
Biological oxygen demand (BOD)		1970 ± 35	977 ± 28	386 ± 14	9 ± 3	7 ± 3	7 ± 3	600
Total suspended solids (TSS)		2037 ± 53	1036 ± 22	401 ± 14	15 ± 2	8 ± 2	5 ± 2	800
Total phosphorus (TP)		8.6 ± 0.4	8.4 ± 0.4	2.36 ± 0.2	0.82 ± 0.1	0.80 ± 0.1	0.80 + 0.1	25
Total nitrogen (TN)		74.2 ± 3.7	72.9 ± 3.6	23.2 ± 3.1	18.5 ± 3.0	16.3 ± 3.0	15.9 ± 3.0	100
Oil		6.93 ± 0.35	0.01 ± 0.001	< 0.001	< 0.001	< 0.001	< 0.001	100
Hydrogen sulfide (H_2_S)		48.9 ± 2.5	35.2 ± 2.1	5.4 ± 0.86	3.9 ± 0.65	3.9 ± 0.65	3.8 ± 0.65	10
Ethylene glycol (EG)		3700 ± 77	688 ± 28	162 ± 13	61 ± 9	52 ± 7	50 ± 6	-
CN		0.025 ± 0.001	0.022 ± 0.001	< 0.001	< 0.001	< 0.001	< 0.001	0.2
Phenols		< 0.00001	< 0.00001	< 0.00001	< 0.00001	< 0.00001	< 0.00001	0.05
Cadmium (Cd)		0.003 ± 0.001	0.003 ± 0.001	< 0.001	< 0.001	< 0.001	< 0.001	0.2
Lead (Pb)		0.001 ± 0.001	0.002 ± 0.001	< 0.001	< 0.001	< 0.001	< 0.001	1
Copper (Cu)		0.009 ± 0.001	0.009 ± 0.001	< 0.001	< 0.001	< 0.001	< 0.001	1.5
Aluminum (Al)		0.092 ± 0.009	0.09 ± 0.009	0.063 ± 0.005	0.11 ± 0.012	0.089 ± 0.008	0.056 ± 0.005	1
Nickel (Ni)		0.056 ± 0.005	0.059 ± 0.005	< 0.001	< 0.001	< 0.001	< 0.001	1
Arsenic (As)		0.027 ± 0.001	0.022 ± 0.001	< 0.001	< 0.001	< 0.001	< 0.001	2
Chromium (Cr)^**^		4.80 ± 0.48	4.28 ± 0.43	2.82 ± 0.35	1.96 ± 0.26	1.96 ± 0.26	1.96 ± 0.26	0.2
Mercury (Hg)		< 0.00001	< 0.00001	< 0.00001	< 0.00001	< 0.00001	< 0.00001	0.2
Tin (Sn)		< 0.00001	< 0.00001	< 0.00001	< 0.00001	< 0.00001	< 0.00001	2
Boron (B)		0.01 ± 0.001	0.01 ± 0.001	< 0.001	< 0.001	< 0.001	< 0.001	1
Colour	Pt/co	683 ± 23	236 ± 15	114 ± 9	55 ± 4	52 ± 4	52 ± 4	
Deposits after 10 min	cm^3^	8	36	3	2	0	0	8
Deposits after 30 min	cm^3^	12	51	5	3	0	0	15

*Limit 93/1962: discharge of wastewater to public sewers.

**Although most parameters met discharge limits, residual chromium concentrations remained above regulatory thresholds, indicating the need for targeted metal-specific polishing in future implementations.

## Cost estimation for the multistage treatment of industrial ethylene glycol (EG) effluent

It is important to clarify that the treated wastewater characteristics reported in Tables 3 and 5 include the effluent quality of the existing industrial wastewater treatment plant, which has been demonstrated to be insufficient for ethylene glycol (EG) removal. Consequently, the proposed multistage treatment system was developed to overcome these limitations through an external solvent-assisted phase separation followed by physicochemical and polishing steps.

A preliminary techno-economic screening was conducted to evaluate the operational feasibility of the proposed treatment scheme. The cost assessment was intentionally designed as an order-of-magnitude estimation, suitable for pilot-scale validation and early-stage technology appraisal, rather than a detailed full-scale industrial economic analysis. All costs were normalized to a functional unit of 1 m³ of treated wastewater, allowing transparent comparison with conventional industrial wastewater treatment approaches.

### Basis and assumptions

The cost estimation was based on experimentally validated treatment performance and pilot-scale operational conditions. The influent wastewater contained approximately 3.7 kg EG m^− 3^, while solvent-assisted phase separation reduced EG concentration to approximately 0.7 kg m^− 3^, indicating substantial removal prior to downstream treatment. For conservative estimation, only partial recovery credit was assigned to the separated EG-rich fraction, accounting for impurities and the need for potential downstream purification.

Dichloromethane (DCM) consumption was calculated based on make-up solvent losses, as solvent recovery efficiencies exceeding 90% were experimentally achieved via rotary evaporation. Energy consumption estimates were derived from standard mixing, pumping, and solvent recovery operations under pilot-scale conditions. Capital expenditure (CAPEX), labor, land, and depreciation costs were excluded to maintain focus on operational feasibility.

### Operating cost components

#### Chemical consumption

Chemical costs included make-up DCM, ferric chloride coagulant, and auxiliary reagents. Coagulant consumption was calculated using the optimized experimental dose (0.75 g L⁻¹), while DCM cost reflected only unrecovered solvent losses after recycling.

#### Energy consumption

Energy requirements were primarily associated with mixing during coagulation, solvent recovery, and filtration operations. Energy demand per cubic meter remained within the range reported for conventional physicochemical treatment systems.

#### Sludge management

Sludge generation was estimated from measured reductions in TSS and coagulant addition. Sludge handling and disposal costs were calculated based on typical industrial disposal rates, acknowledging that sludge volumes remain moderate at the pilot scale.

### Resource recovery consideration

The recovered EG-rich fraction was treated as a resource offset rather than direct revenue. A conservative reuse credit was applied, reflecting scenarios such as internal reuse or blending rather than commercial resale. DCM recovery was considered a cost reduction mechanism rather than a profit stream (Table 6).

### Summary of estimated operating cost

**Table 6:** Preliminary operating cost Estimation for the proposed multistage treatment system.

Cost component	Estimated cost (USD m⁻³)	Contribution (%)
DCM make-up solvent	0.35	28
Coagulant consumption	0.25	20
Energy consumption	0.18	14
Sludge handling & disposal	0.22	18
nZVAl polishing & filtration	0.25	20
Total OPEX	**1.25 USD m** ^**-3**^	**100**
Estimated EG recovery credit	−0.40	—
Net treatment cost	**~ 0.85 USD m** ^**-3**^	—

### Economic scope and limitations

This economic evaluation represents a screening-level analysis intended to demonstrate feasibility rather than provide a definitive industrial cost model. Long-term solvent degradation, labor costs, capital investment, and downstream purification of recovered EG were beyond the scope of this study and should be addressed in future scale-up investigations. Nevertheless, the results demonstrate that integrating solvent-assisted phase separation with conventional treatment and nanomaterial-based polishing does not impose prohibitive operational costs and offers tangible economic advantages through partial resource recovery.

### Environmental and economic benefits

The proposed multistage treatment system addresses the limitations of conventional methods, offering a sustainable and cost-effective solution for the treatment of EG-containing industrial wastewater. It contributes to global efforts to comply with stringent environmental regulations, such as the European Union’s Water Framework Directive and the United States Environmental Protection Agency (EPA) standards^16,48^. The integration of advanced treatment technologies, such as nanotechnology and solvent extraction, represents a step forward in sustainable wastewater management, aligning with the principles of the circular economy^28^.

### Comparison with existing methods

The multistage treatment system proposed in this study outperforms conventional biological treatment methods, which often struggle to completely remove ethylene glycol due to its recalcitrant nature and the formation of toxic intermediates^2^. The combination of physical, chemical, and biological processes in a multistage system offers higher removal efficiencies and greater flexibility in treating complex wastewater matrices^4^. Furthermore, the use of nanotechnology in the final polishing step enhances the overall treatment efficiency, making it suitable for industries with stringent discharge standards^49^.

## Conclusions

This study presents a comprehensive multistage treatment strategy for industrial wastewater containing high concentrations of ethylene glycol (EG), integrating solvent-assisted phase separation, physicochemical treatment, and nanomaterial-based polishing. The proposed framework was developed in response to the demonstrated limitations of the existing industrial wastewater treatment plant, which was unable to achieve effective EG removal or comply with discharge regulations.

The solvent-assisted phase separation step played a critical role in reducing the organic load and enabling partial recovery of an EG-rich fraction. Rather than relying on classical liquid–liquid extraction of free EG, the process was shown to operate through association-driven and co-extraction mechanisms, allowing effective separation of EG-associated organic matter under complex industrial wastewater conditions. This step significantly alleviated the treatment burden on downstream processes.

Subsequent coagulation–precipitation effectively removed suspended solids and colloidal matter, while the tertiary polishing stage employing nano zero-valent aluminum (nZVAl) achieved complete decoloration and further removal of residual soluble organics. Kinetic analysis revealed that the adsorption process followed Avrami-type behavior, indicating a heterogeneous and multistep adsorption mechanism consistent with nanostructured surface interactions.

Pilot-scale implementation of the proposed treatment scheme confirmed its operational feasibility and robustness under real wastewater conditions. The integrated system demonstrated stable performance and reproducibility, supporting its potential applicability as a practical upgrade to conventional industrial treatment configurations.

A preliminary techno-economic screening indicated that the proposed multistage process can be operated at a net treatment cost comparable to conventional high-strength industrial wastewater treatment systems. Solvent recovery and partial EG reuse contributed to reducing operating costs rather than generating direct economic profit, emphasizing the role of resource recovery as a cost-offset mechanism. While the economic evaluation was intentionally conservative and limited to operational feasibility, the results demonstrate that the proposed approach does not impose prohibitive costs and offers a viable balance between treatment efficiency and operational practicality.

Overall, this work advances a treatment/recovery framework that shifts the focus from single-step pollutant degradation toward integrated process design. By combining solvent-assisted separation, conventional physicochemical treatment, and nanotechnology-based polishing, the proposed system provides a scalable and adaptable solution for EG-laden industrial effluents. Future studies should address long-term operation, detailed capital cost analysis, and optimization of solvent and resource recovery pathways to support full-scale industrial implementation.

## Data Availability

All data generated or analyzed during this study are included in this published article.

## References

[1] Khan, A. et al. Equilibrium adsorption studies of some aromatic pollutants from dilute aqueous solutions on activated carbon at different temperatures. *J. Colloid Interface Sci.* **194** (1), 154–165 (1997). 9367594 10.1006/jcis.1997.5041

[2] Mantzavinos, D. et al. Enhancement of biodegradability of industrial wastewaters by chemical oxidation pre-treatment. *J. Chem. Technol. Biotechnology: Int. Res. Process. Environ. Clean. Technol.* **79** (5), 431–454 (2004).

[3] Yang, Q. et al. Optimal integration design of sustainable ethylene glycol production process considering economic benefit and environmental impact*.* 396: p. 136540. (2023).

[4] Chen, J. et al. Physical–Chemical–Biological Pretreatment for Biomass Degradation and Industrial Applications:* A Review*. in *Waste*. MDPI. (2024).

[5] Al-Salihi, S. A. et al. Synthetic Biology: Pioneering the Next Bio Revolution for a sustainable planet. (2024). 10.1093/jambio/lxaf202 40833632

[6] Noor, M. H. M., N.J.J.o.W, P. E. & Ngadi Global research landscape on coagulation-flocculation for wastewater treatment: A 2000–2023 bibliometric analysis*.* 64: p. 105696. (2024).

[7] Li, C. et al. Treatment of high-salinity organic wastewater by advanced oxidation processes: Research progress and prospect*.* 60: p. 105272. (2024).

[8] Mantzavinos, D. & Kalogerakis, N. J. E. Treatment of Olive mill effluents: part I. Organic matter degradation by chemical and biological processes—an overview. Environment international, **31**(2): pp. 289–295. (2005). 10.1016/j.envint.2004.10.005 15661297

[9] Pignatello, J. J. et al. Advanced oxidation processes for organic contaminant destruction based on the Fenton reaction and related chemistry. Critical reviews in environmental science and technology. **36**(1): pp. 1–84. (2006).

[10] Nidheesh, P. J. R. A. Heterogeneous Fenton catalysts for the abatement of organic pollutants from aqueous solution: a review. *RSC Adv.* **5** (51), 40552–40577 (2015).

[11] Srivastava, R. R. et al. Challenges, regulations, and case studies on sustainable management of industrial waste. *Minerals* **13** (1), 51 (2022).

[12] Van der Bruggen, B. & Vandecasteele, C. J. E. Removal of pollutants from surface water and groundwater by nanofiltration: overview of possible applications in the drinking water industry. 122(3): pp. 435–445. (2003). 10.1016/s0269-7491(02)00308-1 12547533

[13] Chaturvedi, A. et al. A comprehensive review on the integration of advanced oxidation processes with biodegradation for the treatment of textile wastewater containing Azo dyes. *Rev. Chem. Eng.* **38** (6), 617–639 (2022).

[14] Plan, C. & Belgium For a cleaner and more competitive Europe. 28. (2020).

[15] Plant, H. W. T. The US Environmental Protection Agency (EPA) Proposes to Reissue a National Pollutant Discharge Elimination System (NPDES) Permit to Discharge Pollutants Pursuant to the Provisions of the Clean Water Act (CWA) to.

[16] Sciences, N. A. et al. Improving the EPA Multi-Sector General Permit for Industrial Stormwater Discharges (National Academies, 2019).

[17] LU, S. et al. Progress and prospect on monitoring and evaluation of united nations SDG 6 (Clean water and Sanitation). *Target* **36** (8), 904–913 (2021).

[18] European Commission, E. Directive 2000/60/EC of the European Parliament and of the Council of 23 October 2000 Establishing a framework for community action in the field of water policy. *EC Directive*. **327** (43), 1–72 (2000).

[19] Farag, R. S., Elshfai, M. M. & Mahmoud, A. S. Adsorption and kinetic studies using nano zero valent iron (nZVI) in the removal of chemical oxygen demand from aqueous solution with response surface methodology and artificial neural network approach. *J. Environ. Biotechnol. Res.* **7** (2), 12–22 (2018).

[20] Jason, A. et al. AOP Performance at Wastewater Treatment Plants. in. Proc. 2018 Annual AIChE Meeting, Pittsburgh, PA, (October 28 – November 2)*.* (2018).

[21] Mahmoud, A. S. et al. Nano zero-valent aluminum (nZVAl) preparation, characterization, and application for the removal of soluble organic matter with artificial intelligence, isotherm study, and kinetic analysis. *Air Soil. Water Res.* **12**, 1178622119878707 (2019).

[22] Rabie, S. et al. Green Synthesis of Nano Iron Carbide: Preparation, Characterization and Application for Removal of Phosphate from Aqueous Solutions. in Proc. 2018 Annual AIChE Meeting, Pittsburgh, PA, (October 28 – November 2)*.* (2018).

[23] Harter, T. Groundwater Quality and Groundwater Pollution (UCANR, 2003).

[24] Philander, S. G. Encyclopedia of Global Warming and Climate Change (Sage, 2008).

[25] PEDDI, P. K. Pilot-scale demonstration of hzvi process for treating (Texas A&M University, 2011).

[26] Wijesekara, S. et al. Fate and Transport of Selection Nutrients and Heavy Metals in Nanoscale Zero Valent Iron Amended Sand Columns. (2014).

[27] Jahan, N. et al. A comprehensive review on the sustainable treatment of textile wastewater: zero liquid discharge and resource recovery perspectives*.* 14(22): p. 15398. (2022).

[28] Macarthur, E. & Heading, H. J. E. M. F. How Circular Econ. Tackles Clim. Change **1**: 1–71. (2019).

[29] Rabie S. Farag, M.M.E., Ahmed S. Mahmoud, Mohamed K. Mostafa, Ahmed Karam, and R.W. Peters. *S*tudy the Degradation and Adsorption Processes of Organic Matters from Domestic Wastewater using Chemically Prepared and Green Synthesized Nano Zero-Valent Iron. in 2019 Annual AIChE Meeting; Orlando, FL, November 10–15, 2019. 2019. https://www.researchgate.net/publication &#8230.

[30] Baird, R.B., Standard methods for the examination of water and wastewater,* 23rd*. 2017, Water Environment Federation, American Public Health Association, American&#8230.

[31] Maha M. Elshfai, A.S.M. and M.A. Elsaid. Comparative Studies of using nZVI and Entrapped nZVI in Alginate Biopolymer (Ag/ nZVI)for Aqueous Phosphate Removal. in 12th international conference on nano-technology for green and sustainable construction. 2021. https://www.researchgate.net/publication &#8230.

[32] Zeldowitsch, J. Über Den mechanismus der Katalytischen oxydation von CO an MnO2. *Acta Physicochim URSS*. **1**, 364–449 (1934).

[33] Avrami, M. Kinetics of phase change. II transformation-time relations for random distribution of nuclei. *J. Chem. Phys.* **8** (2), 212–224 (1940).

[34] Ho, Y. S. & McKay, G. Kinetic models for the sorption of dye from aqueous solution by wood. *Process Saf. Environ. Prot.* **76** (2), 183–191 (1998).

[35] Ho, Y. S. & McKay, G. Pseudo-second order model for sorption processes. *Process Biochem.* **34** (5), 451–465 (1999).

[36] Fola, A. T., Idowu, A. A. & Adetutu, A. Removal of Cu2 + from aqueous solution by adsorption onto quail eggshell: kinetic and isothermal studies. *J. Environ. Biotechnol. Res.* **5** (1), 1–9 (2016).

[37] Mahmoud, A. S. et al. Isotherm and kinetic studies for heptachlor removal from aqueous solution using Fe/Cu nanoparticles, artificial intelligence, and regression analysis. Sep. Sci. Technol*.*, 55 (4), 684-696. (2020)

[38] Mahmoud, M. et al. Comparison of aluminum and iron nanoparticles for chromium removal from aqueous solutions and tannery wastewater, empirical modeling and prediction. Emergent Mater., 5 (6), pp.1729-1744. (2022)

[39] Tomaszewski, K., Szymanski, A. & Lukaszewski, Z. J. T. Separation of Poly (ethylene glycols) into fractions by sequential. *liquid–liquid Extr.* **50** (2), 299–306 (1999). 10.1016/s0039-9140(99)00024-7 18967721

[40] Tratnyek, P. G. And R.L.J.N.t. Johnson. Nanotechnologies Environ. Cleanup. **1** (2), 44–48 (2006).

[41] Phenrat, T. et al. Aggregation and sedimentation of aqueous nanoscale zerovalent iron dispersions. *Environ. Sci. Technol.* **41** (1), 284–290 (2007). 17265960 10.1021/es061349a

[42] Fu, F. & Wang Q.J.J.o.e.m. Remov. Heavy Metal Ions Wastewaters*: *Rev. **92**(3): 407–418. (2011). 10.1016/j.jenvman.2010.11.011 21138785

[43] Mahmoud, A. S. et al. Innovative Decentralized Wastewater Treatment: Coupling Coagulation, nZVAl Adsorption, Sand Filtration, and ANN-Based Optimization. 18: p. 11786221251392205. (2025).

[44] Mahmoud, M. et al. Comparison of aluminum and iron nanoparticles for chromium removal from aqueous solutions and tannery wastewater. *Empir. Model. Prediction*. **5** (6), 1729–1744 (2022).

[45] Mahmoud, A. S., Farag, R. S. & Elshfai, M. M. J. E. J. P. Reduction of organic matter from municipal wastewater at low cost using green synthesis nano iron extracted from black tea: Artificial intelligence with regression analysis. 29(1): pp. 9–20. (2020).

[46] El-Shafei, M. et al. Effects of entrapped nZVI in alginate polymer on BTEX removal. in AIChE Annual Meeting. San Francisco, CA. (2016).

[47] Mahmoud, M. & A.S.J.E.m. Mahmoud Wastewater treatment using nano bimetallic iron/copper, adsorption isotherm, kinetic studies, and artificial intelligence neural networks. *Emergent Mater.* **4** (5), 1455–1463 (2021).

[48] Lanz, K. & Scheuer, S. EEB handbook on EU water policy under the Water Framework Directive. (2001).

[49] Mahmoud, A. S. et al. Effective chromium adsorption from aqueous solutions and tannery wastewater using bimetallic Fe/Cu nanoparticles: response surface methodology and artificial neural network. 14: p. 11786221211028162. (2021).

